# MicroRNA and lncRNA as the Future of Pulmonary Arterial Hypertension Treatment

**DOI:** 10.3390/ijms24119735

**Published:** 2023-06-04

**Authors:** Łukasz Wołowiec, Martyna Mędlewska, Joanna Osiak, Anna Wołowiec, Elżbieta Grześk, Albert Jaśniak, Grzegorz Grześk

**Affiliations:** 1 Department of Cardiology and Clinical Pharmacology, Collegium Medicum in Bydgoszcz, Nicolaus Copernicus University, 87-100 Toruń, Poland; lordtor111@gmail.com (Ł.W.);; 2 Department of Geriatrics, Division of Biochemistry and Biogerontology, Collegium Medicum in Bydgoszcz, Nicolaus Copernicus University, 87-100 Toruń, Poland; anna.wolowiec@cm.umk.pl; 3 Department of Pediatrics, Hematology and Oncology, Collegium Medicum in Bydgoszcz, Nicolaus Copernicus University, 87-100 Toruń, Poland; ellag@cm.umk.pl

**Keywords:** pulmonary arterial hypertension, PAH, epigenetics, drug targets, long non-coding RNA, lncRNA, microRNA, miRNA

## Abstract

Pulmonary hypertension (PH) is characterized by a progressive increase in pulmonary arterial pressure and pulmonary vascular resistance. In a short time, it leads to right ventricular failure and, consequently, to death. The most common causes of PH include left heart disease and lung disease. Despite the significant development of medicine and related sciences observed in recent years, we still suffer from a lack of effective treatment that would significantly influence the prognosis and prolong life expectancy of patients with PH. One type of PH is pulmonary arterial hypertension (PAH). The pathophysiology of PAH is based on increased cell proliferation and resistance to apoptosis in the small pulmonary arteries, leading to pulmonary vascular remodeling. However, studies conducted in recent years have shown that epigenetic changes may also lie behind the pathogenesis of PAH. Epigenetics is the study of changes in gene expression that are not related to changes in the sequence of nucleotides in DNA. In addition to DNA methylation or histone modification, epigenetic research focuses on non-coding RNAs, which include microRNAs (miRNAs) and long non-coding RNAs (lncRNAs). Preliminary research results give hope that targeting epigenetic regulators may lead to new, potential therapeutic possibilities in the treatment of PAH.

## 1. Introduction

Pulmonary hypertension (PH) is an abnormal increase in pressure in the pulmonary artery that can occur in the course of diseases of the heart, lungs and pulmonary vessels. It is defined by an increase in mean pulmonary artery pressure (mPAP), as determined by right heart catheterization. With the advent of the new ESC guidelines from 2022, the definition of PH has been changed. According to the new assumptions, PH is defined as mPAP >20 mmHg at rest, not >25 mmHg as previously [1,2]. It is estimated that this disease occurs in 1% of the population, and the leading causes are left heart disease and lung diseases [3]. However, the etiology varies by population—for example, in Asia, compared to the West, there is a higher rate of congenital heart defects—and PH-related connective tissue diseases [4]. The progress of PH is associated with increased mortality—despite treatment, mortality remains at a high level [3,5]. The incidence of PH increases with age [6]. The lack of effective treatment and multiple co-morbidities in the elderly result in a deterioration in the quality of life. Elderly patients have worse quality of life, and what is more, most of them show symptoms of depression [7,8]. Symptoms of PH include exercise-induced shortness of breath, excessive tiredness, shortness of breath when bending forward (bendopnea), palpitations, exercise-induced abdominal bloating and nausea, weight gain due to fluid retention, and fainting (during or shortly after exercise). Rare symptoms caused by pulmonary artery dilatation include exertional chest pain (associated with dynamic compression of the left main coronary artery) and hoarseness (caused by compression of the left recurrent laryngeal nerve—i.e., cardiovocal syndrome or Ortner’s syndrome) [1,9,10].

There are five groups of hypertension: group 1—pulmonary arterial hypertension (PAH); group 2—pulmonary hypertension in the course of left heart disease; group 3—pulmonary hypertension in the course of lung diseases and/or hypoxia; group 4—chronic thromboembolic pulmonary hypertension; and group 5—pulmonary hypertension with an unclear and/or multifactorial mechanism [11]. In this review, we will focus on PAH. It is a disease based on increased proliferation and resistance to apoptosis of cells in the small pulmonary arteries. PAH is a disease leading to approximately 15% mortality within 1 year of diagnosis, and in 2012, PAH mortality was 51% within 7 years of diagnosis [12,13]. PAH is a rare but progressive and currently incurable disease. Looking at its pathogenesis and the constant search for new therapeutic possibilities is an important process in the process of searching for new, more effective therapeutic possibilities [14,15].

## 2. Current Therapeutic Options

One group of drugs currently used in patients with PAH are calcium channel blockers (CCBs). The first studies showing the potential benefits of their use date back to the 1970s [16,17]. CCBs inhibit the influx of calcium into vascular cells, leading to smooth muscle cell relaxation and vasodilation. As mentioned earlier, small pulmonary artery spasm is considered a component of the pathogenesis of PAH, which makes treatment with CCB seem rational in this situation. Currently, CCB is recommended for patients who respond positively to pulmonary artery responsiveness testing, as only these patients may benefit from treatment [1,18]. A study by Rich et al. found that in a group of 64 patients diagnosed with PAH and treated with CCBs, mPAP and pulmonary vascular resistance (PVR) decreased sharply by 15% and 26%. It has also been noticed that survival was improved in that group of patients over five years of observation [19]. In patients with idiopathic PAH (IPAH), congenital PAH (HPAH), or drug or toxin-associated PAH (DPAH), high doses of CCBs are recommended (class 1 recommendation). Unfortunately, the group that benefits from therapy with CCB is quite small—people with a long-term CCB treatment response account for <10% of patients with IPAH [20]. During reassessment, additional vascular reactivity testing should be performed to detect a sustained vascular response to support a possible increase in CCB dose [1].

Another group of drugs used in the treatment of PAH are endothelin receptor antagonists (ERAs). Endothelin is an endogenous peptide released from the vascular endothelium, binding to A and B receptors and showing vasoconstrictive activity [21]. Receptor blockade causes blood vessels to dilate. This group of drugs includes ambrisentan—a drug that preferentially blocked endothelin A receptors in the ARIES study [22]. In addition to ambrisentant, this group includes two other drugs—Bosentan and Macitentan—both of them being non-selective blockers of A and B receptors. Their effect has been proven in randomized clinical trials [1,23].

Another drug target is the nitric oxide (NO) pathway. The importance of NO was discovered in the 1970s. NO is colorless gas and also an important cellular signaling molecule which participates, for example, in vasodilation and smooth muscle relaxation [24]. Phosphodiesterase 5 (PDE5) is the enzyme that controls the nitric oxide (NO)-dependent production of intracellular cyclic guanosine monophosphate (cGMP). Guanylate cyclase is an enzyme that synthesizes cGMP by catalyzing the GTP–cGMP reaction. The inhibition of PDE5 is commonly used as an element in the multi-compound therapy of PH [25]. PDE5 inhibitors, by their mechanism of action, dilate blood vessels by blocking PDE5 in the smooth muscle cells lining the blood vessels. PDE5 inhibitors include Sildenafil and Tadalafil. Their effect has been demonstrated in many studies [1].

Soluble guanylyl cyclase activators may be a promising therapeutic option in the pharmacotherapy of PH. Based on the CHEST-1 and PATENT-1 trials, riociguat was introduced into clinical practice for treating chronic thromboembolic pulmonary hypertension (CTEPH) [26]. Riociguat works in two ways: it sensitizes guanylate cyclase to endogenous NO, and it also stimulates guanylate cyclase directly, independently of NO. Its effectiveness has been demonstrated, among others, in improving exercise capacity [27].

**Figure 1:**
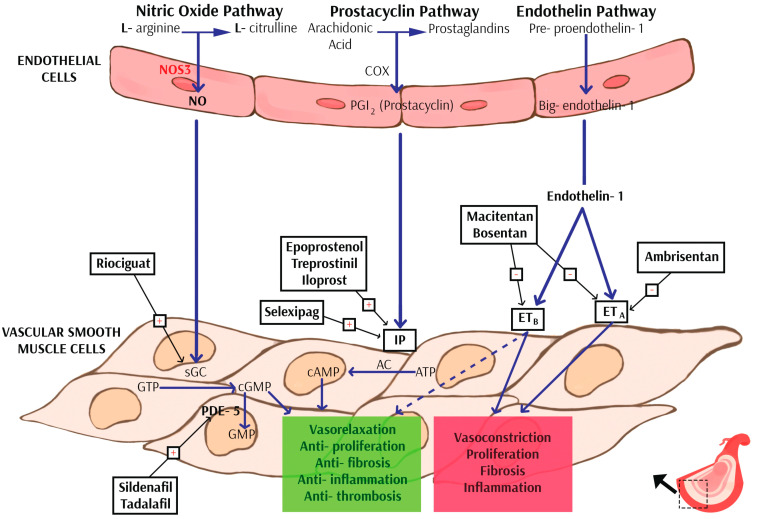
Three major signaling pathways involved in the pathogenesis of pulmonary arterial hypertension and the mechanism of action for contemporary drugs. The dashed line from ETB denotes action of endothelial ETB activation via NO and PGI2 production. Explanation of abbreviations: NOS3—nitric oxide synthase 3; NO—nitric oxide; COX—cyclooxygenase; sGC—soluble guanylate cyclase; GTP—guanosine-5’-triphosphate; cGMP—cyclic guanosine monophosphate; GMP—guanosine monophosphate; PDE-5—phosphodiesterase type 5; AC—adenylate cyclase; AMP—adenosine monophosphate; ATP—adenosine triphosphate; IP—prostaglandin I receptor; ETa/b—endothelin receptor a/b.

In addition to the above-mentioned drugs, prostacyclin analogues and prostacyclin receptor agonists—epoprostenol, iloprost, treprostinil, beraprost and selexipag—are also used in the treatment of PAH [1,2]. As can be seen from the above-mentioned groups of drugs, their actions are based mainly on three pathways involved in the pathogenesis of PAH: the endothelin (ET), nitric oxide (NO), and prostacyclin pathways [22,24,26] (Figure 1).

## 3. Pathophysiology and Molecular Background of PAH

PAH can develop as a result of various pathological states and factors, such as connective tissue diseases, congenital heart defects, HIV infection, medications, or genetic factors [11]. However, all of these multiple causes initiate similar pathophysiological mechanisms. Pulmonary artery endothelial cell (PAEC) defects are among the most fundamental processes underlying PAH and trigger a cascade of molecular events, leading to development of the disease [28]. PAEC dysfunction leads to an imbalance between vasodilatory and vasoconstrictive factors, favoring the latter. Furthermore, endothelial damage results in enhanced production of thrombotic factors, inflammatory cytokines and other mediators that affect pulmonary artery smooth muscle cells (PASMCs), thus promoting the proliferation and migration of PASMCs and myofibroblasts to the endothelial cells layer and disturbing PASMC apoptosis [29,30]. All of the above-mentioned alterations deteriorate the ability of the pulmonary arteries (PAs) to dilate, therefore stimulating the fibrosis and pathological remodeling of arterial walls, which ultimately generates increased blood pressure and vascular resistance in PAs [29].

However, the currently available treatment does not affect vascular remodeling and degeneration. It is therefore crucial to thoroughly understand the complex molecular and biological backgrounds of PAH if we want to develop effective, targeted therapies in the future [31]. Among the most significant pathological processes underlying PAH are disturbances in the endothelin-1-dependent pathways, as well as in the prostacyclin-mediated pathways; decreased bioavailability of nitric oxide; dysfunction of ionic calcium and potassium channels; mitochondrial metabolism disorders; and disturbances in the renin–angiotensin–aldosterone axis; and genetic abnormalities, including mutations in the bone morphogenetic protein receptor type 2 (BMPR 2) gene and epigenetic alterations [32]. It is precisely on the latter that we will focus our attention on in this article.

## 4. Epigenetic Mechanisms of Pathogenesis in PAH

Epigenetics is the science that deals with the inherited mechanisms of gene expression that are not dependent on changes in the DNA sequence but are dependent on changes in the chromosome [33]. Epigenetic mechanisms can regulate gene expression through chemical modifications of DNA bases and changes in the chromosomal superstructure in which the DNA is packaged. The dominant epigenetic mechanisms are DNA methylation, chromatin modifications and non-coding RNAs [34]. As is known, epigenetic modifications may be acquired de novo or may be inherited [35]. These changes, on the one hand, can contribute to genetic diversity, and on the other hand, they often lead to developmental abnormalities and diseases. The disruption of gene expression patterns may affect, among others, the development of autoimmune diseases, cancer, and other disease entities—including PAH [36]. Animal models indicate that these mechanisms play a fundamental role in the development of inflammation, obesity, diabetes, atherosclerosis and cardiovascular disease. Abnormal DNA methylation and histone modifications were found in atherosclerotic studies [37]. Additionally, it has recently been reported that a small interfering RNA targeting the proprotein convertase sutilin/kexin type 9 can promote hepatic steatosis and hepatocellular carcinoma via upregulation [38]. Due to the discovered epigenetic mechanisms, research on epigenetics is currently under intensive development, as the reversibility of epigenetic changes is a potential target for new therapeutic strategies.

**Figure 2:**
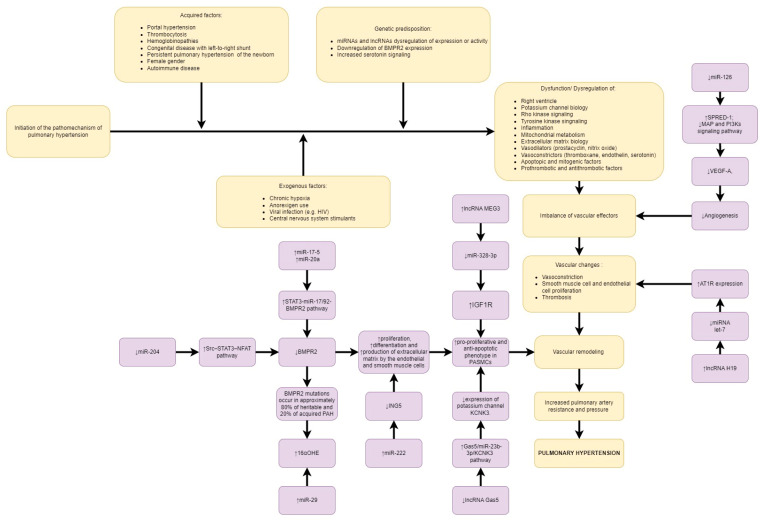
PAH pathomechanisms, including the influence of microRNA and lncRNA. Explanation of abbreviations: BMPR2—bone morphogenetic protein receptor type 2; SRC—proto-oncogene tyrosine-protein kinase Src; STAT3—signal transducer and activator of transcription 3; NFAT—nuclear factor of activated T-cells; 16αOHE—16α-hydroxyestrone; ING5—inhibitor of growth family member 5; MEG3—maternally expressed gene 3; IGF1R—insulin-like growth factor 1 receptor; PASMCs—pulmonary artery smooth muscle cells; GAS5—growth arrest-specific transcript 5; KCNK3—potassium two pore domain channel subfamily K member 3; AT1R—angiotensin II type 1 receptor; VEGF-A—vascular endothelial growth factor A; SPRED-1—sprouty-related, EVH1 domain-containing protein 1; MAP—mitogen-activated protein; PI3Ks—phosphatidylinositol-3-kinase; LET-7—lethal-7.

The epigenetic hypothesis of PAH consists of changes in DNA methylation levels at the superoxide dismutase 2 (SOD2) and granulysin (GNLY) gene loci; histone H1 levels; abnormal expression levels of histone deacetylases (HDACs) and bromodomain-containing protein 4 (BRD4); and dysregulated microRNA networks (miRNAs) [39,40]. Discussion of all these mechanisms is beyond the scope of this article; therefore, in this paper, we focus on discussing the role of non-coding RNA in the development of PAH. Unlike mRNAs, ncRNAs are not encoded for proteins or peptides and include siRNAs, microRNAs (miRNAs), long non-coding RNAs (lncRNAs), circular RNAs (circRNAs), and extracellular RNAs [41]. An example of a substance used in cardiology whose mechanism is based on siRNA is inclisiran, a drug used in hypercholesterolemia [42,43]. Non-coding RNA seems to be a very promising topic—based on research in recent years, it has been reported that the normalization of several types of miRNAs inhibits PH [44,45]. The molecular pathways leading to the development of PAH are presented in Figure 2.

## 5. Role of miRNAs and lncRNAs in PAH

In vertebrae, over 50% of protein coding mRNA is controlled by miRNA [46]. The expression of proteins is silenced by miRNA binding to specific mRNAs, thus playing a fundamental role in the post-transcriptional regulation of gene expression [41,45]. Different miRNAs participate in the processes of differentiation, apoptosis, and cell proliferation; hence, the dysregulation of the expression or activity of these RNAs results in a disturbance in cellular homeostasis, which consequently can lead to numerous diseases, such as PAH [47]. It is known that miRNA malfunctions trigger enhanced proliferation of endothelial, smooth muscle, and adventitial cells in PAs [48]. Currently, mounting new evidence linking various miRNAs and their altered expression—either increased or decreased—with PAH pathogenesis is emerging. The vast array of publications based on various experimental methods makes it challenging to identify the most significant miRNAs in terms of PAH pathogenesis and potential therapeutic targets.

In 2020, Ferreira et al. reviewed the available literature and made an attempt to identify the miRNAs that could possibly have the biggest clinical potential. Using specifically designed analytical tools, they distinguished four miRNAs which showed an aberrant pattern of expression compared to healthy cells, both in animal and human models, to investigate the pathogenesis of PAH, namely, miR-29, miR-124, miR-140, and miR-204 [48].

Courboulin et al. proved that miR-204 is downregulated in pulmonary artery smooth muscle cells (PASMCs) coming from patients with PAH, as well as from rats with induced PAH. The research has shown that the decreased expression of miR-204 in PASMCs induces the activation of the Src–STAT3–NFAT pathway, thus promoting the pro-proliferative and anti-apoptotic phenotype in PASMCs [49]. Moreover, this path is also associated with the aforementioned BMPR2 protein, whose role in PAH pathogenesis cannot be overestimated. The mutations in the BMPR2 gene are responsible for approximately 80% of cases of familial PAH; they are also found in around 20% of cases of sporadic PAH [50]. Loss of function of the BMPR2 protein or a decrease in its activity and the disturbance of its signaling pathways is a key and permissive event in the whole process of PAH pathogenesis—this also affects the change in phenotype in favor of anti-apoptotic and pro-proliferative ones [51,52]. BMPR2 plays a significant role in the proliferation, differentiation, and production of extracellular matrix by the endothelial and smooth muscle cells [53]. In their previously mentioned work, Courboulin et al. found that a decrease in miR-204 expression can indirectly affect and downregulate BMPR2 expression. Their research has revealed a rise in BMPR2 expression in PASMCs obtained from patients with PAH and rats with induced PAH treated with miR-204 mimics. These findings may be attributed to the inhibition of the signal transducer and activator of transcription 3 (STAT3) by miR-204, the upregulation of which leads to a consequent reduction in BMPR2 expression [53].

In a study conducted by Brock et al., it was demonstrated that increased levels of interleukin 6 (Il-6), which is engaged in PAH development, caused an upregulation in the expression of the miRNA cluster miR-17/92 [54]. Il-6 stimulates the expression of miR-17/92 through the STAT3 signaling pathway. Two molecules, miR-17-5 and miR-20a, appeared to be the key molecules coded by the above-mentioned RNA cluster, which directly interfere with BMPR2 mRNA and block it. Therefore, the overexpression of these miRNAs leads to the downregulation of BMPR2 in PAECs in patients with PAH.

Another RNA molecule, miR-124, is yet another RNA molecule that contributes to PAH pathogenesis, which has drawn scientists’ attention. Wang et al. have proven that reduced expression of miR-124 in pulmonary artery adventitial fibroblasts initiates epigenetic reprogramming, hence modifying the cells’ phenotypes and leading to enhanced proliferation, migration, and increased inflammation [55]. A different study analyzed a subpopulation of blood outgrowth endothelial cells (BOECs) and PAECs obtained from patients with PAH. Caruso et al. have demonstrated that these cells exhibit low expression of miR-124, which in turn results in an increase in the concentration of polypyrimidine-tract-binding protein 1 (PTBP1). On the other hand, the upregulation of PTBP1 leads to a switch in expression from the normally expressed pyruvate kinase M1 (PKM1) to the M2 isoform (PKM2) of the enzyme. This change is associated with excessive proliferation and metabolism based on aerobic glycolysis (the Warburg effect) [56]. The Warburg effect has been primarily described in cancer cells; however, it is also observed in the cells of PAH patients. It favors extensive growth and promotes excessive proliferation and apoptosis resistance [57].

In their research, Chen et al. investigated the correlation between miR-29 upregulation and elevated levels of the estrogen metabolite 16α-hydroxyestrone (16αOHE) in the pathogenesis of heritable PAH (HPAH), associated with BMPR2 mutation. In the experiment, the scientists used transgenic mice with a BMPR2 mutation along with cells obtained in an autopsy from the lungs of female patients with HPAH. It has been confirmed that females with HPAH caused by BMPR2 mutation exhibit an estrogen metabolism dysfunction, which eventually leads to elevated levels of 16αOHE [58]. Additionally, a study conducted by Austin et al. showed that elevated levels of 16αOHE are a permissive factor and increase the risk of HPAH onset. Chen et al. revealed that it is associated with miR-29 upregulation, which causes a disruption in mitochondrial structure and activity and, therefore, aberrant cells’ energetic metabolisms. This also results in molecular damage, which in turn leads to insulin resistance, among others [58,59].

Interestingly, miR-126 has also captured the interest of researchers. It is an endothelial specific miRNA that raises the levels of vascular endothelial growth factor A (VEGF-A) and thus stimulates a proangiogenic response in endothelial cells (ECs). The scientists observed a 60% decrease in miR-126 expression in the cells obtained from vastus lateralis muscle from patients with PAH, compared to healthy individuals. This decrease led to an upregulation of miR-126 target protein SPRED-1 (sprouty-related, EVH1 domain-containing protein 1), which is a VEGF inhibitor. This cascade of events, triggered by a reduction in miR-126 expression, results in a loss of microcirculation in skeletal muscles and impaired angiogenesis, ultimately contributing to the exercise intolerance of patients with PAH [60].

Moreover, another study conducted by Potus et al. has proven that the downregulation of miR-126 in cardiomyocytes of PAH patients decreases micro-vessel density and thus promotes the decompensation of right ventricle failure in PAH. The decrease in miR-126 concentration elevates the levels of SPRED-1, which in turn represses mitogen-activated protein kinase (MAP) and the phosphatidylinositol-3-kinase signaling pathway and suppresses angiogenesis [61].

Two molecules, miR-126 along with miR-143/145, were the subjects of a different research carried out by Bockmeyer et al. [62]. Plexiform lesions (PLs) are one of the most common pathomorphological findings in PAs remodeled in the course of PAH. PLs are complex, glomeruloid-like vascular formations that develop in thickened pulmonary arterial walls. In addition, concentric lesions (CLs) are other aberrant structures that form in the process of concentric laminar fibrosis and hypertrophy of media cells in Pas [63]. It was revealed that the miR-126 levels were significantly elevated in ECs in PLs compared to the cells in CLs. In the latter cells, on the other hand, the expression of miR-143/145 was upregulated, in contrast to PLs cells. Nevertheless, the concentration of miR-126 was considerably decreased in PL cells, as well as in CLs, compared to healthy controls. The aim of the authors was to demonstrate the pivotal miRNA molecules involved in the development of diverse histological PAH manifestations [62]. Key studies are presented in Table 1.

## 6. Role of lncRNAs in PAH

In recent years, the attention of researchers has not only been captured by the miRNAs being key elements to thoroughly comprehending PAH pathogenesis and potential therapeutic targets. Long noncoding RNAs (lncRNAs) are also identified among the epigenetic mechanisms engaged in PAH development. Even though lncRNAs do not code any protein in the vast majority of cases, there is evidence that some of them may carry information about micro- and polypeptides [64,65]. Specific lncRNAs exhibit diverse subcellular locations—the majority of these molecules can be found in the nucleus and fewer in the cytoplasm. On the contrary, some of them are found in both of the above-mentioned locations [66]. Due to this fact, lncRNA molecules can effectively regulate gene expression on different levels, from epigenetic DNA modifications and transcriptional changes to modulating mRNA stability and translational or post-translational control [66,67]. Additionally, lncRNAs can act as competing endogenous RNAs (ceRNA), thus blocking miRNA and regulating mRNA expression [68]. Nevertheless, the role of lncRNA in PAH development remains not fully explained. Existing evidence has shown that the dysregulation of these molecules leads to the dysfunction of PASMCs and PAECs in the course of PAH [69].

A study conducted by Su et al. demonstrated that lncRNA H19 is upregulated in lung cells obtained from rats and mice with monocrotaline (MCT)-induced PAH. The elevation in H19 levels results from platelet-derived growth factor BB (PDGF-BB) stimulation, which is one of many inflammatory cytokines involved in PAH development. The increase in H19 concentration leads to enhanced expression of type 1 receptor for angiotensin II (AT_1_R) in PASMCs, thereby promoting cell proliferation and favoring vasoconstriction in response to angiotensin II. The elevated H19 levels silence miRNA let-7b, which directly upregulates AT_1_R expression. On the contrary, the H19 gene knockout decreased the percentage of muscularized vessels and was a protective factor in PAH rodents [70].

Interestingly, two independent studies investigating the role of lncRNA MEG3 in PAH pathogenesis have resulted in quite opposite outcomes. In the research carried out by Sun et al., the group of scientists analyzed the expression of MEG3 in pulmonary cells from PAH patients and in the cells from healthy individuals. The study showed a significant downregulation of MEG3 expression in PASMCs derived from patients with PAH. Furthermore, experimental silencing of the MEG3 gene led to enhanced proliferation and migration of human PASMCs, thereby demonstrating the influence of low lncRNA MEG3 expression in PAH pathogenesis [71]. However, in 2019, Xing et al. analyzed the role of the aforementioned lncRNA in hypoxia-induced PAH (HPH) and discovered that MEG3 levels were substantially elevated in PASMCs. In this experiment, the researchers have used mice, initially exposed to hypoxia to induce HPH, as well as PASMCs from patients with idiopathic PAH. The study has shown that MEG3 upregulation was triggered by hypoxia-inducible factor (HIF), particularly by the HIF1a. The excessively produced MEG3 sequestrates and thus lowers the concentration of miR-328-3p, which in turn leads to increased expression of insulin-like growth factor 1 receptor (IGF1R) [72]. Consequently, IGF1R affects the cells’ growth, differentiation, survival, and migration [73]. In summary, the upregulation of lncRNA MEG3 expression stimulates proliferation and reduces apoptosis in PASMCs. Nonetheless, here arises the question of how two such seemingly comparable studies can lead to fundamentally divergent conclusions. Xing et al. propose a few hypotheses that may explain the inconsistent results. Firstly, the cause may lie in the choice of transcriptional variants of lncRNA MEG3, as there are 15 of them. Secondly, the authors of the publication point to the fact that MEG3 levels were elevated specifically in hypoxia-stimulated PASMCs. However, MEG3 was downregulated in the other tissues, including lung cells, except from PASMCs. This finding may suggest a tissue- or even cell-specific expression of this particular lncRNA [72].

A study conducted by Hao et al. focused on lncRNA Gas5 and its influence on rats with hypoxia-induced PAH and hypoxia-stimulated human PASMCs (hPASMCs). In the analyzed cells, a decreased expression of Gas5 was observed. The researchers proved that this lncRNA is a competitive inhibitor of miR-23b-3p, which in turn blocks the expression of potassium channel KCNK3. Complete lncRNA Gas5 silencing in the examined cells resulted in excessive proliferation and migration of hPASMCs. This study has shown that the Gas5/miR-23b-3p/KCNK3 axis is one of many mechanisms behind PAH development, and hence presents as one of the potential future therapeutic targets [74].

On the other hand, Han et al. assessed the role of reduced concentration of lncRNA CASC2 in hypoxia-stimulated PASMCs [75]. CASC2 is renowned as a tumor suppressor [76]. The scientists performed a number of bioinformatic analyses and experiments, revealing that CASC2 is a significant factor in PAH pathogenesis by competitively binding to miR-222 and acting as a ceRNA. Downregulated CASC2 stimulates the abnormal expression of miR-222, which in turn suppresses the inhibitor of growth 5 (ING5), causing excessive proliferation and migration in PASMCs [75]. Key studies are presented in Table 2. 

## 7. Therapeutic Potential of miRNAa and lncRNAs

Currently, the available therapies act only symptomatically and do not target the underlying etiology of the disease; thus, the treatment results are still unsatisfactory, and PAH remains a condition with high morbidity and mortality rates. Therefore, a thorough understanding of all the molecular mechanisms participating in PAH development is essential if we want to successfully treat this disease. In the past few years, mounting evidence has been reported demonstrating the critical role of miRNAs and lncRNAs in PAH pathogenesis. Consequently, further research in this field is necessary to explore the potential for the development of novel drugs.

In the previously mentioned study, Courboulin et al. performed an experimental treatment on rats with MCT-induced PAH. The animals received synthetic miR-204 molecules through intratracheal nebulization, which resulted in pulmonary arterial blood pressure reduction, along with a decrease in the thickness of pulmonary arterial walls and the right ventricle wall. Moreover, the scientists observed a significant decline in the activation of the p-STAT3–NFATc2 pathway, leading to a decrease in proliferation and apoptosis resistance in PASMCs. It is also worth mentioning that miR-204 expression was also decreased in the buffy coat, which indicates its value as a potential biomarker of PAH [49].

An exceptionally interesting study conducted by Lee et al. analyzed the influence of exosomes on mice with hypoxia-induced PAH and human PAECs (hPAECs). The exosomes are secreted membrane microvesicles, 30–100 nm in size, used in intercellular communication via paracrine signaling. In this particular study, the scientists used exosomes derived from mesenchymal stromal cells (MSC), so-called MEXs (MSC-derived exosomes), which were isolated from mice bone marrow and human umbilical cords and Wharton’s jelly. Following the treatment with MEXs, a rise in the concentration of miR-204 was observed, a molecule that is primarily downregulated in the cells of PAH patients. Furthermore, MEX therapy inhibited the STAT3 pathway, which is known to stimulate the miR-17/92 cluster and simultaneously block miR-204 expression. Due to this novel treatment, the researchers managed to target some of the molecular pathways and mechanisms underlying PAH, including those associated with miRNA dysfunction [77].

In their study, Caruso et al. transfected the investigated cells with miR-124 molecules and detected a decline in glycolysis and in lactic acid concentration to control levels. Moreover, the transfection resulted in proliferation rate normalization [56].

The aim of an experiment performed by Chen et al. was to regulate the miR-29 levels in PASMCs obtained from transgenic mice with HPAH, caused by a BMPR2 mutation. For six weeks, the rodents received injections with anti-miR-29 (amiR-29). The treatment resulted in a significant reduction in right ventricle systolic pressure and PVR. Furthermore, it reversed the increased muscularization seen in mice with HPAH. The administration of amiR-29 also positively affected the mice PASMCs’ condition on a molecular level by diminishing their insulin resistance and improving their mitochondrial morphology, which is impaired in the course of HPAH [58].

Potus et al. successfully restored normal angiogenesis in the analyzed cells by increasing miR-126 concentration. In a research work from 2014, not only did they reverse the impaired angiogenesis in endothelial cells from skeletal muscles, but they also increased the microcirculation density by transfecting the examined cells with miR-126 mimic [60]. Moreover, in a similar study from 2015, the scientists managed to restore vascular density in cardiomyocytes, which were obtained from PAH patients, using miR-126 injections. Intravenous administration of miR-126 mimic also proved to be beneficial for rats with MCT-induced PAH. After two weeks of such treatment, the researchers observed an improvement in right ventricle function and cardiac output on echocardiography, along with an increase in exercise capacity of the rodents [61].

On the other hand, the potential of lncRNAs as therapeutic targets or disease biomarkers still remains almost unexplored, as scientists have to overcome multiple technical challenges already during the early stages of planning an experiment. It is due to the unique qualities of these molecules that the problems arise. Firstly, in the majority of cases, there is lack of evolutionary conservation of lncRNAs’ nucleotide sequences between humans and species of animals used in experiments. As a result, designing the preclinical phase of a study becomes difficult. Moreover, in such cases, it would be complicated to compare the results between the species and to draw conclusions applicable to humans [69]. Furthermore, most of the lncRNA molecules can be expressed as different transcript variants, which makes research on their biological function and mechanisms of action even more challenging [78]. Additionally, the majority of lncRNAs are active in the nucleus, which creates a mechanical barrier and hinders the use of antisense oligonucleotides or RNA interference mechanisms in the research [66].

However, Xing et al., in a successful attempt to silence the expression of lncRNA MEG3, used specifically designed liposomes in order to deliver complementary siRNA that binds to the target molecules of the investigated cells. The R8-Lip-siMEG3 system was precisely created to transport the drug directly to the lung cells of mice used in this experiment. The administration of the siRNA silenced MEG3 expression, which in turn reduced the expression of Ki-67—a protein associated with cell proliferation. Moreover, it led to PVR reduction and a decrease in right ventricle systolic pressure [72].

A fresh perspective on developing novel therapies was also brought by Han et al. in their study, which proved that increased expression of lncRNA CASC2, resulting in miR-222 downregulation, suppresses the proliferation and migration of PASMCs [75].

Nonetheless, it is important to underline the fact that most of the above-mentioned studies on the use of miRNA and lncRNA as future therapeutic targets are not free from limitations. Clearly, one such limitation is the early stage the research is in and the use of cell models. The cells stimulated in an artificial environment to induce the PAH phenotype do not fully mimic the complex, PAH-causing mechanisms in the human body. Furthermore, in cell-based trials, the therapeutic substances can be administered directly into the cell. It is therefore crucial to develop efficient drug delivery systems, designed to act in the lungs of PAH patients. Last but not least, the animal models, however helpful and necessary, are not perfect; therefore, the results of animal-based studies cannot be directly applied to humans. Therefore, we still need thorough research and clinical trials to push forward the development of novel PAH treatments.

## 8. Circular RNA (circRNA)

CircRNA is another molecule belonging to the group of non-coding RNAs that may be a potential therapeutic target in PH. CircRNA is a newly identified type of non-coding RNA molecule with a unique closed-loop structure. CircRNAs have been reported to coordinate gene expression by acting as miRNA sponges, interacting with RNA-binding proteins (RBPs), and modulating transcription [79]. CircRNA is more stable than linear RNA [80]. This molecule was discovered about 50 years ago [81]. However, it was described for the first time in human organisms in 2012, which initiated further research [82]. To date, around 30,000 circRNAs have been found in human tissues [83]. It has been shown recently that circRNA can be a prognostic marker in cardiovascular diseases. In one study, an association between the level of expression of circRNA (so-called MICRA—myocardial infarction-associated circular RNA) and left ventricular dysfunction due to myocardial infarction was noticed [84].

As mentioned above, increased proliferation and resistance to apoptosis of pulmonary vascular cells in small pulmonary arteries are key pathological components of pulmonary vascular remodeling and the onset of PH. Many types of circRNA have been found, among others, in plasma and lung tissues. They may be involved in the regulation of proliferation and apoptosis of PAECs and PASMCs, leading to pulmonary vascular remodeling. One possible mechanism underlying this phenomenon is that circRNAs can regulate the function of PAECs and PASMCs by acting as miRNA sponges. However, other potential mechanisms determining the role of circRNA in the pathophysiology of PH are still being actively investigated [85]. Ongoing research will certainly reveal the exact role of circRNA over time, which may provide an answer to whether it can be used as a target or therapeutic method.

## 9. Summary

The development of epigenetics may give hope to patients with PAH, for whom effective therapeutic methods are still not available. The use of non-coding RNAs is one of the potential promising therapeutic strategies. It is possible that in the future, innovative diagnostic tests and treatment regimens will be based on epigenetic mechanisms and they will be incorporated into medical practice. At the moment, we have to be satisfied with the currently available treatment, but maybe the next few years will see a breakthrough and an opportunity for PAH patients.

## Figures and Tables

**Table 1:** Research on miRNAs in PAH.

Authors	Year	Carried out on Animal/Human Cell Cultures	Comments
Brock M, Trenkmann M, Gay RE, et al. [54]	2009	Cell cultures (human cells)	Described a novel STAT3–miR-17/92–BMPR2 pathway, thus providing a mechanistic explanation for the loss of BMPR2 in the development of PH. Possible overexpression of miR-17-5 and miR-20a leads to downregulation of BMPR2 in PAEC.
Wang D, Zhang H, Li M, et al. [55]	2014	Cell cultures (human and animal cells)	Described that reduced expression of miR-124 in pulmonary artery adventitial fibroblasts initiates epigenetic reprogramming, hence modifying the cells’ phenotypes and leading to enhanced proliferation, migration, and increased inflammation.
Chen X, Talati M, et al. [58]	2016	Cell cultures (human and animal cells)	Investigated the correlation between miR-29 upregulation and elevated levels of the estrogen metabolite 16α-hydroxyestrone in pathogenesis of heritable PAH (HPAH), associated with BMPR2 mutation.
Potus, F., Malenfant, S., et al. [60]	2014	Cell cultures (human and animal cells)	Demonstrated that a cascade of events triggered by a reduction in miR-126 expression results in a loss of microcirculation in skeletal muscles and impaired angiogenesis, ultimately contributing to exercise intolerance of patients with PAH.

PAEC—pulmonary artery endothelial cells; PAH—pulmonary artery hypertension; PH—pulmonary hypertension.

**Table 2:** Research on lncRNAs in PAH.

Authors	Year	Carried out on Animal/Human Cell Cultures	Comments
Su H, Xu X, et al. [70]	2018	Cell cultures (animal cells)	Demonstrated that lncRNA H19 is upregulated in lung cells obtained from rats and mice with monocrotaline induced PAH. The elevation in H19 levels results from platelet-derived growth factor BB stimulation, which is one of many inflammatory cytokines involved in PAH development. The increase in H19 concentration leads to enhanced expression of type 1 receptor for angiotensin II in PASMCs, thereby promoting cell proliferation and favoring vasoconstriction in response to angiotensin II. The elevated H19 levels silence miRNA let-7b, which directly upregulates AT1R expression.
Sun Z, Nie X, et al. [71]	2017	Cell cultures (human cells)	Described that upregulation of lncRNA MEG3 expression stimulates proliferation and reduces apoptosis in PASMCs.
Hao X, Li H, et al. [74]	2020	Cell cultures (animal and human cells)	Demonstrated that Gas5 (lncRNA) is a competitive inhibitor of miR-23b-3p, which in turn blocks the expression of potassium channel KCNK3. Complete lncRNA Gas5 silencing in the examined cells resulted in excessive proliferation and migration of hPASMCs. The Gas5/miR-23b-3p/KCNK3 axis is one of many mechanisms behind PAH development.
Han Y, Liu Y, et al. [75]	2020	Cell cultures (human cells)	Described that downregulated CASC2 stimulates the abnormal expression of miR-222, which in turn suppresses the inhibitor of growth 5 (ING5), causing excessive proliferation and migration in PASMCs

lncRNA—long non-coding RNA; PAH—pulmonary artery hypertension; PASMCs—pulmonary artery smooth muscle cells.

## Data Availability

Not applicable.
